# Image quality comparison of lower extremity CTA between CT routine reconstruction algorithms and deep learning reconstruction

**DOI:** 10.1186/s12880-023-00988-6

**Published:** 2023-02-19

**Authors:** Daming Zhang, Chunlin Mu, Xinyue Zhang, Jing Yan, Min Xu, Yun Wang, Yining Wang, Huadan Xue, Yuexin Chen, Zhengyu Jin

**Affiliations:** 1 Department of Radiology, State Key Laboratory of Complex Severe and Rare Disease, Peking Union Medical College Hospital, Chinese Academy of Medical Sciences and Peking Union Medical College, Bejing, 100730 China; 2 Department of Radiology, Beijing Sixth Hospital, Beijing, 100007 China; 3 Canon Medical Systems, Beijing, 100015 China; 4 Department of Vascular Surgery, State Key Laboratory of Complex Severe and Rare Disease, Peking Union Medical College Hospital, Chinese Academy of Medical Sciences and Peking Union Medical College, Beijing, 100730 China

**Keywords:** Tomography, Angiography, Deep learning, Algorithms

## Abstract

**Background:**

To evaluate the image quality of lower extremity computed tomography angiography (CTA) with deep learning–based reconstruction (DLR) compared to model-based iterative reconstruction (MBIR), hybrid-iterative reconstruction (HIR), and filtered back projection (FBP).

**Methods:**

Fifty patients (38 males, average age 59.8 ± 19.2 years) who underwent lower extremity CTA between January and May 2021 were included. Images were reconstructed with DLR, MBIR, HIR, and FBP. The standard deviation (SD), contrast-to-noise ratio (CNR), signal-to-noise ratio (SNR), noise power spectrum (NPS) curves, and the blur effect, were calculated. The subjective image quality was independently evaluated by two radiologists. The diagnostic accuracy of DLR, MBIR, HIR, and FBP reconstruction algorithms was calculated.

**Results:**

The CNR and SNR were significantly higher in DLR images than in the other three reconstruction algorithms, and the SD was significantly lower in DLR images of the soft tissues. The noise magnitude was the lowest with DLR. The NPS average spatial frequency (f_av_) values were higher using DLR than HIR. For blur effect evaluation, DLR and FBP were similar for soft tissues and the popliteal artery, which was better than HIR and worse than MBIR. In the aorta and femoral arteries, the blur effect of DLR was worse than MBIR and FBP and better than HIR. The subjective image quality score of DLR was the highest. The sensitivity and specificity of the lower extremity CTA with DLR were the highest in the four reconstruction algorithms with 98.4% and 97.2%, respectively.

**Conclusions:**

Compared to the other three reconstruction algorithms, DLR showed better objective and subjective image quality. The blur effect of the DLR was better than that of the HIR. The diagnostic accuracy of lower extremity CTA with DLR was the best among the four reconstruction algorithms.

## Background

As one of the most common modalities for evaluating lower extremity PAD, runoff computed tomography angiography (CTA) has become a robust noninvasive imaging routine due to its short acquisition time, high spatial resolution, and increased anatomical coverage of the whole vascular tree [1, 2]. CTA truly revolutionized the diagnostic approach to vascular disorders of the lower extremities thanks to the high temporal resolution with fast execution time, the coverage of long vascular territories in a few seconds, the acquisition of isotropic datasets with the possibility of different reconstruction approaches, and accurate visualization of the anatomy and pathological abnormalities to allow appropriate treatment planning [3]. As an imaging modality based on X-rays, the balance between image quality and radiation dose is an important consideration [4]. Another problem in lower extremity CTA is the influence of image quality on small vessel visualization [5]. Thus, the CT reconstruction algorithm used can be an important factor for image quality.

The CT reconstruction algorithm was developed from filtered back projection (FBP) to hybrid iterative reconstruction (HIR) and model-based iterative reconstruction (MBIR); although it improved the image quality, diagnostic performance, and reduction of radiation dose in many clinical applications of CTA [6, 7], there are still some remaining unsatisfactory aspects in image texture, spatial resolution, and lesion detectability. Recently, a new deep learning-based CT reconstruction (DLR) algorithm (Advanced Intelligent Clear-IQ Engine [AiCE], Canon Medical Systems Corporation) has become commercially available. It was trained to differentiate noise from signals with the deep convolutional neural network (DCNN) by using MBIR patient datasets acquired with high tube current as golden standard clinical reference images [8]. The DLR reconstruction process begins in the raw data domain which is modified based on the detailed scanner model information, this raw data is then reconstructed to form a seed image for the DCNN. After that, the input image is analyzed by several network layers. The neurons in the network layer learn what features to look for based on the training data. DLR improves image quality with lower image noise, better sharpness, and more accurate diagnostic performance on the phantom study, cerebral CT, chest CT, coronary CTA, and abdominal CT [8–13]. AiCE is not the only available deep learning-based reconstruction algorithm, as there is also True Fidelity (GE Healthcare System), which has been applied in the lower extremity vessels study with better quantitative image quality for DLR in comparison with HIR [14]. Based on the promising results of previous studies, we speculate that its application to runoff CTA can improve image quality and diagnostic performance.

The purpose of this study was to evaluate the image quality of vessels and soft tissue in lower extremity runoff CTA of DLR compared to HIR, MBIR, and FBP, and to assess the diagnostic performance of runoff CTA using the DLR reconstruction algorithm.

## Methods

### Patient population

This was a retrospective study. The review board of our institution approved the study (HS-2427) and waived the requirement for informed consent due to its retrospective status.

Fifty-five consecutive patients who underwent runoff CTA with the indication given by the vascular surgeon were recorded, and 5 patients were excluded for metal implants. Fifty patients who had undergone runoff CTA between January 22, 2021, and May 27, 2021, were included.

### CT acquisition parameters

All scans were performed on a 320-row-detector CT scanner (Aquilion One GENESIS Edition, Canon Medical Systems Corporation, Japan). The scan was performed from the distal abdominal aorta to the toes in a craniocaudal direction.

Ninety milliliters of iodinated contrast agent (370 mgI/mL, Iopromide, Ultravist, Bayer Health care, Germany) at a flow rate of 4 mL/second was administered intravenously. A region of interest (ROI) was placed on the distal abdominal aorta. When the predefined threshold of 150 HU was reached, 6 s later the scan automatically initiated.

The scanning parameters included slice collimation: 80 × 0.5 mm, rotation time: 0.75 s, pitch: 0.637, automatic modulation tube current: 40–200 mA (with noise index SD = 10), and tube voltage: 100 kVp.

The volumetric CT dose index (CTDIvol) and dose-length product (DLP) were automatically saved for each examination. The effective radiation dose was estimated using the DLP multiplied by the conversion factor of 0.0058 at 100 kV reported by Saltybaeva et al. [15].

### Image reconstruction

All acquisitions were reconstructed using four reconstruction techniques: DLR (AiCE Body Sharp), HIR (AIDR 3D, kernel FC08), MBIR (FIRST Body Sharp), and FBP (kernel FC08). All reconstructions had a slice thickness of 1 mm and an interval of 0.8 mm. The field of view was set to 400.4 mm with a pixel matrix of 512 × 512.

### Digital subtraction angiography (DSA)

Twenty-one patients received the DSA procedure within 30 days after CTA. DSA was performed on an angiographic system (Axiom Artis, Siemens Healthcare). The pelvic, thigh, and lower leg arteries of the symptomatic leg were examined with 5 ml iodinated contrast medium (320 mg I/ml iodixanol; Visipaque; GE Healthcare) per segment (aorta-iliac, femoral-popliteal and below the knees) using the stepping DSA. The posteroanterior projections were captured first and the left and right anterior oblique projections were added if the stenosis could not be assessed from the posteroanterior projection. Lesions were considered significant with visual luminal narrowing of ≥ 50%.

### CT image analysis

#### Quantitative analysis

**Fig. 1:**
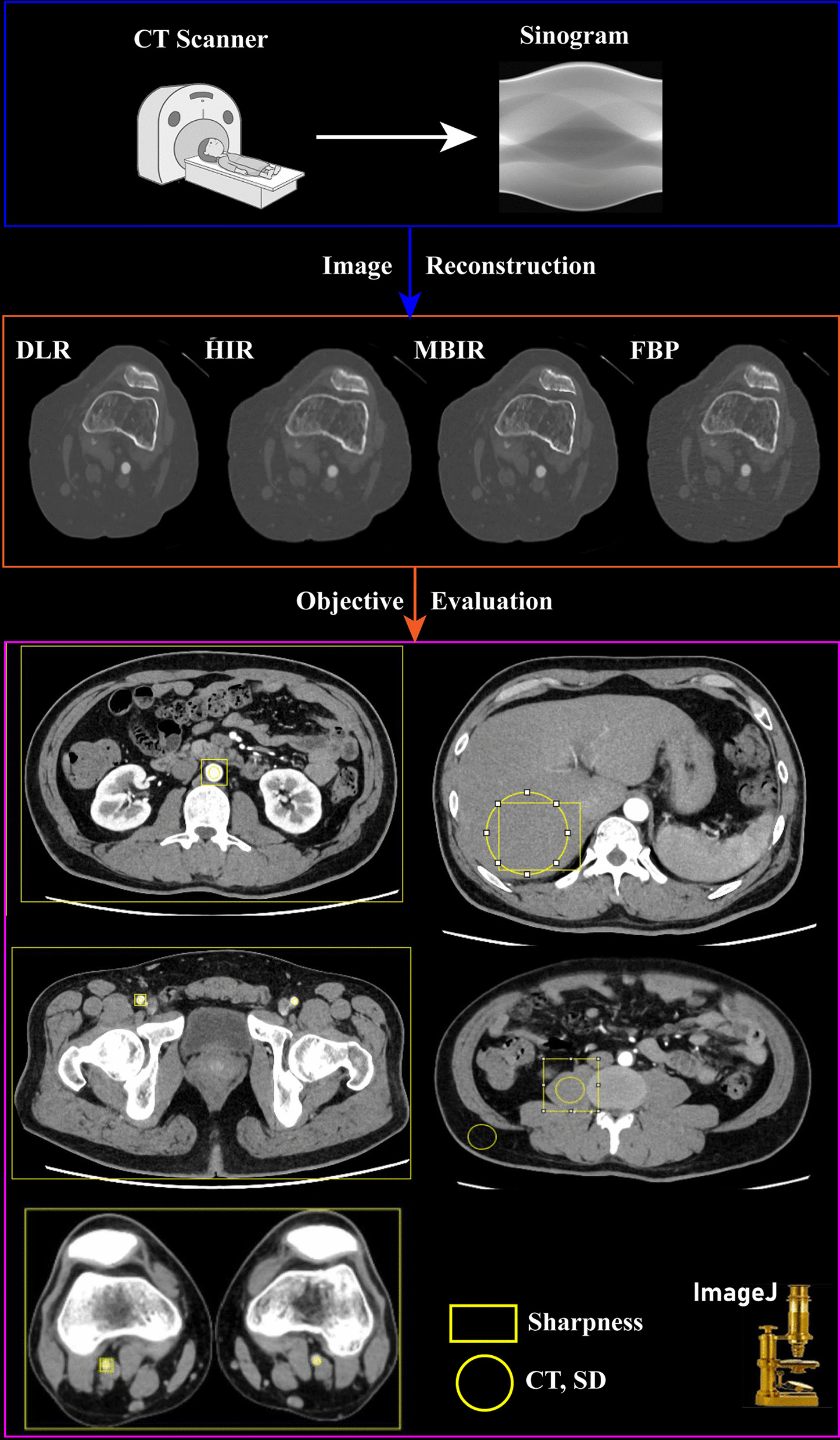
Flow chart of quantitative analysis

One senior radiologist (with 10 years of experience) drew the vessels’ region of interest (ROI) (aorta, common femoral artery, and popliteal artery), liver, and psoas muscle based on DLR images. One medical physicist retrieved the CT attenuation and standard deviation (SD) of the ROIs using ImageJ (V1.53a, National Institutes of Health, Bethesda, Maryland) and MATLAB (R2014) from the same locations of HIR, MBIR, and FBP images (Fig. 1). Then, the contrast-to-noise ratio (CNR) and signal-to-noise ratio (SNR) were calculated as follows: CNR = (HU_target _− HU_fat_)/SD_fat_ and SNR = HU_target_/SD_target_, respectively.

Noise power spectrum (NPS) curves [16–18] of three different anatomical structures (liver, the aorta, and the psoas muscle) were derived from each patient’s CT images by using imQuest software (Version 7.1, Duke University, Durham NC). Measurements were performed on 10 consecutive slices to reduce the influences of noise and artifacts, and ROIs were carefully placed in the homogeneous region to guarantee the accuracy of the measured data. The normalized NPS curves were calculated by dividing the NPS value by the maximum value of the corresponding NPS.

In addition, the blur effect which represents objective sharpness was also calculated using MATLAB. The method was introduced in a study conducted by Chankue Park et al. The “blur metric” program (Do Quoc Bao (2022). Image Blur Metric (https://www.mathworks.com/matlabcentral/fileexchange/24676-image-blur-metric)) was used to evaluate the level of image sharpness as a non-reference quantitative value [19]. Before the blur metric value calculation, one radiologist drew eight rectangular ROIs including five anatomic structures and three whole-slice sections using ImageJ (Fig. 1). The five anatomic structures included the aorta, femoral artery, popliteal artery, liver, and muscle; the three whole-slice sections included the aorta, femoral artery, and popliteal artery. A scientist retrieved the blur metric data of the ROI area for each image (DLR, MBIR, Hybrid-IR, and FBP). The blur metric program quantifies sharpness by blurring the input image, and the variation between adjacent pixels is analyzed. First, intensity variations of CT attenuation between neighboring pixels are analyzed. Second, the low-pass-filtered image is used to measure the intensity variations between pixels. Third, a comparison between original and filtered images is made to evaluate the degree of blurring. The original image is believed to be sharp when there is a large difference in intensity variation. In the end, the blur metric value ranges from 0 to 1, with lower scores denoting sharper images and higher scores representing blurrier images.

#### Qualitative analysis

**Fig. 2:**
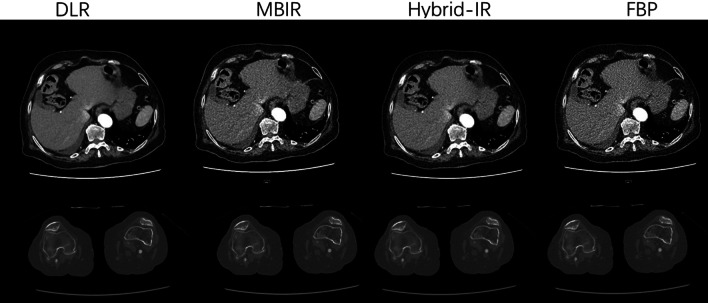
Example slice demonstrating the impact of the different reconstruction algorithms for the runoff CTA of the liver with a window setting of the soft tissue and popliteal artery with a window setting of the bone

The subjective image quality based on image noise, subjective sharpness, and natural appearance was independently evaluated by two radiologists with 10 years and 3 years of experience, respectively, (Fig. 2) with the window setting of bone (width 2000 HU; level 350 HU) and soft tissue (width 350 HU; level 40 HU). A 5-point Likert scale was used, as detailed in Table 1.

**Table 1:** Description of the categories of image quality characteristics

Image quality characteristic	Score				
	1	2	3	4	5
Noise	Excessive noise, impairs diagnostic quality	Substantial noise increase; reduced image quality	Moderate increase of noise compared to current standard	Average noise; equal to current standard	Low noise magnitude, lower than current standard
Subjective sharpness	Excessive blurring, impairs diagnostic quality	Substantial blurring; reduced image quality	Moderate sharpness; less sharpness compared to current standard	Average sharpness; equal to current standard	Sharp delineation of structures, superior to current standard
Natural appearance	Unnatural appearance impairing diagnostic quality	Substantial unnatural appearance, moderate impairment of diagnostic quality	Moderate unnatural appearance, not impairing diagnostic quality	Natural appearance is normal, equal to current standard	Natural appearance is superior to current standard

### Statistical analysis

R software (version 3.6.1; http://www.R-project.org) was used to perform the statistical analysis. The Shapiro–Wilk test was used to determine the normality of the data distribution. For continuous variables with a normal distribution, one-way repeated analysis of variance (ANOVA) was used, and paired samples t-tests with Bonferroni correction were used for subsequent multiple comparisons. For data with nonnormal distribution, the Friedman test was applied, and the Wilcoxon signed rank test with Bonferroni correction was applied for multiple comparisons. *p* < 0.05 was considered statistically significant. Interreader reliability was assessed using a Kendall rank correlation coefficient. The diagnostic accuracy, such as sensitivity, specificity, and positive and negative predictive values, for the detection of significant stenosis, was calculated with DSA serving as a reference standard. The comparison of sensitivity and specificity of four reconstruction algorithms was calculated using McNemar’s test. *p* < 0.05 was considered statistically significant.

## Results

Fifty patients were enrolled in the study (38 males, mean age 59.8 ± 19.2 years, range 19–91 years; mean body mass index (BMI) 24.1 ± 3.0 kg/m^2^, range 18.4–31.2 kg/m^2^). 60 slices of CT images were used for analysis in one patient, and 3000 slices of CT images in total were analyzed in this study. Among them, 21 patients (18 males, mean age 69.2 ± 9.6 years, range 49–91 years; mean BMI 24.1 ± 2.3 kg/m^2^, range 20.1–27.0 kg/m^2^) underwent clinically indicated DSA. The CTDIvol was 3.7 ± 0.8 mGy, and the DLP was 500.0 ± 119.5 mGycm. The radiation dose was 2.9 ± 0.7 mSv.

### Quantitative analysis

The results of the quantitative assessment of the image qualities (CT, SD, SNR, and CNR) are summarized in Table 2.

**Table 2:** Quantitative analysis

Parameter	DLR	MBIR	HIR	FBP	p*
*Aorta*					
HU	488.70 ± 105.57	462.89 ± 95.56*	481.51 ± 103.93*°	488.44 ± 104.49°˜	0.55
SD	17.42 ± 12.89	32.08 ± 9.89*	25.63 ± 13.42*°	53.06 ± 10.86*°˜	< 0.001
SNR	35.65 ± 13.94	15.24 ± 4.28*	21.50 ± 7.57*°	9.56 ± 2.95*°˜	< 0.001
CNR	48.28 ± 11.12	25.07 ± 5.60*	33.42 ± 8.62*°	15.84 ± 4.41*°˜	< 0.001
*Femoral artery*					
HU	491.40 ± 142.18	446.60 ± 167.26*	451.23 ± 172.31*°	460.10 ± 178.17*°˜	< 0.001
SD	32.42 ± 26.44	35.73 ± 23.36*	38.17 ± 25 ± 25*	51.98 ± 22.27*°˜	< 0.001
SNR	23.10 ± 14.79	15.60 ± 8.69*	15.49 ± 11.30*	9.91 ± 5.00*°˜	< 0.001
CNR	47.24 ± 15.04	24.03 ± 8.66*	31.12 ± 11.85*°	15.00 ± 6.38*°˜	< 0.001
*Popliteal artery*					
HU	402.64 ± 170.65	377.33 ± 160.73*	367.05 ± 154.54*°	374.93 ± 159.45* ˜	< 0.001
SD	43.90 ± 40.21	41.15 ± 37.73	41.58 ± 31.86	47.64 ± 33.59*°˜	< 0.001
SNR	15.80 ± 12.33	14.86 ± 14.86	12.89 ± 8.87*	10.99 ± 8.60*°˜	< 0.001
CNR	39.64 ± 15.47	20.47 ± 8.1*	25.87 ± 10.53*°	12.44 ± 5.58*°˜	< 0.001
*Liver*					
HU	66.55 ± 9.12	66.94 ± 9.03	66.22 ± 9.59	67.91 ± 9.65*°˜	< 0.001
SD	11.32 ± 2.59	28.41 ± 3.52*	18.90 ± 2.39*°	49.46 ± 10.20*°˜	< 0.001
SNR	6.11 ± 1.41	2.40 ± 0.52*	3.56 ± 0.78*°	1.40 ± 0.27*°˜	< 0.001
CNR	13.95 ± 2.53	7.58 ± 1.50*	9.72 ± 1.99*°	4.54 ± 0.88*°	< 0.001
*Psoas muscle*					
HU	57.79 ± 8.05	58.24 ± 8.06	57.61 ± 7.98°	59.58 ± 8.01*°˜	< 0.001
SD	10.46 ± 1.34	24.88 ± 3.36*	17.52 ± 2.29*°	43.91 ± 6.02*°˜	< 0.001
SNR	5.67 ± 1.36	2.40 ± 0.54*	3.37 ± 0.83*°	1.38 ± 0.29*°˜	< 0.001
CNR	13.22 ± 2.32	7.18 ± 1.40*	9.21 ± 1.81*°	4.32 ± 0.87*°˜	< 0.001

*DLR* deep learning–based reconstruction, *MBIR* model-based iterative reconstruction, *HIR* hybrid-iterative reconstruction, *FBP* filtered back projection

Pairwise comparisons showed significant differences from DLR (*), MBIR (°), and HIR (˜) (*p* < 0.05)

Compared to MBIR and HIR, DLR showed higher CT attenuations of the aorta (all *p* < 0.001). DLR images of the femoral artery and popliteal artery showed the highest CT attenuation compared with other images (all *p* < 0.001). For the liver and psoas muscle, FBP showed significantly different CT attenuation with other reconstructions (all *p* < 0.001), while DLR showed similar CT attenuations with HIR and MBIR (all *p* > 0.001).

For image noise, DLR showed the lowest noise of arteries imaged (aorta, femoral artery, and popliteal artery), followed by MBIR, HIR, and FBP (all *p* < 0.001). There was no significant difference between the HIR and MBIR in the femoral and popliteal arteries (*p* = 0.27 and *p* = 0.42, respectively). No significant differences were observed between DLR and HIR, DLR and MBIR, or HIR and MBIR (*p* = 1, *p* = 0.22, *p* = 0.42, respectively). For the liver and psoas muscle, DLR also showed the lowest noise, and FBP had the highest noise (all *p* < 0.001).

The SNR of the DLR in all structures was significantly higher than those of the other three types of images (all *p* < 0.001), except in the popliteal artery, where there was no significant difference between the DLR and MBIR in the popliteal artery (*p* = 1). In the aorta, the trend of SNR from low to high was FBP, MBIR, and HIR and was significantly different with pairwise comparison (all *p* < 0.001). In the femoral artery and popliteal artery, FBP showed the lowest SNR, while there was no significant difference between MBIR and HIR (*p* = 1, *p* = 0.55, respectively). In addition, there was no significant difference between the DLR and MBIR in the popliteal artery (*p* = 1).

Similar to the SNR, the CNR of the DLR in all structures was significantly higher than those of the other three types of images (all *p* < 0.001). The trend of CNR in all structures from low to high was FBP, MBIR, and HIR in all structures and was significantly different from each other with pairwise comparison (all *p* < 0.001).

**Fig. 3:**
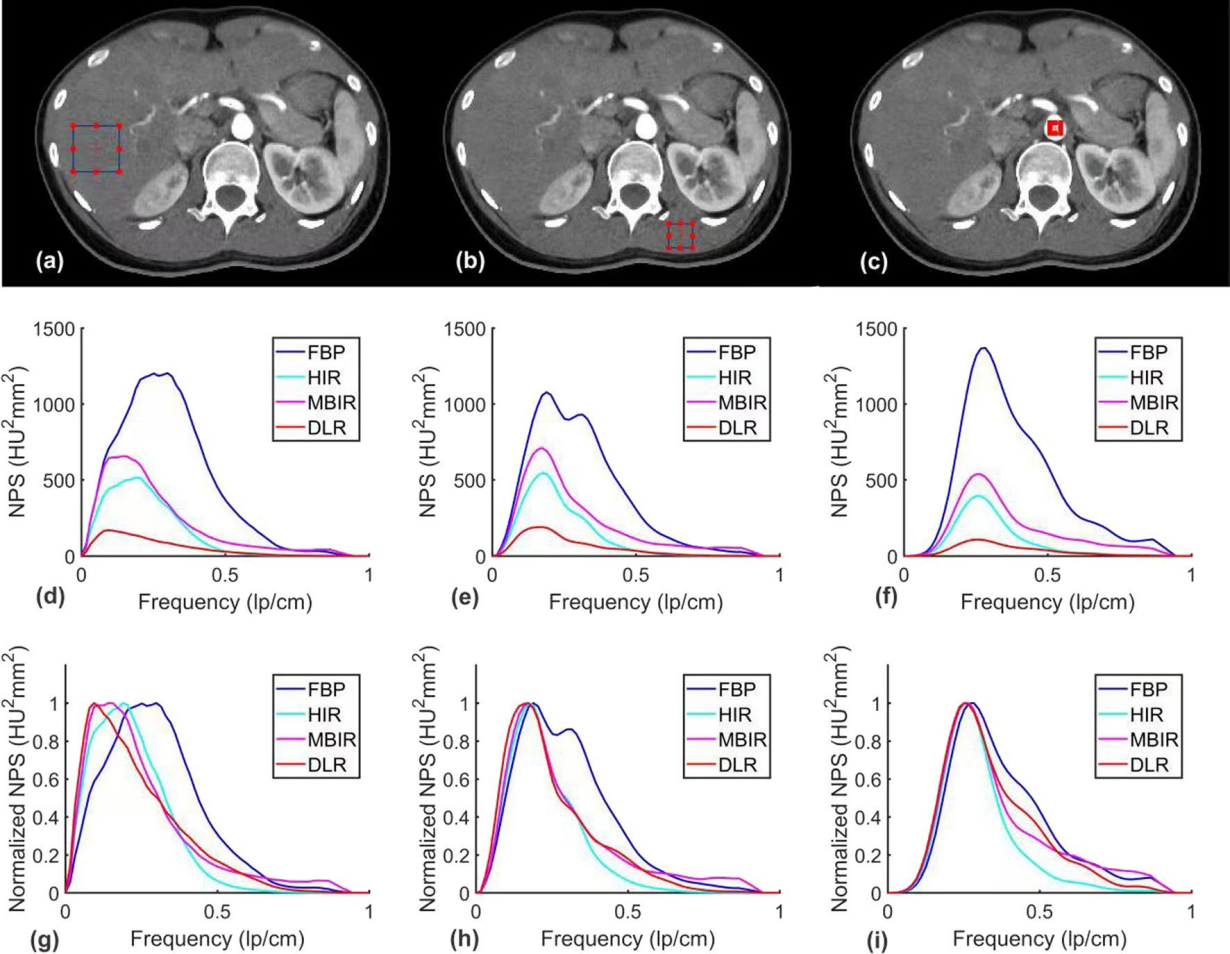
Axial images with ROIs in red are placed over different anatomical structures (**a**, **b**, **c**). NPS curves (**d**, **e**, **f**) and normalized NPS curves (**g**, **h**, **i**) were obtained for all reconstruction algorithms. **a**, **d**, **g** demonstrate the liver, **b**, **e**, **h** demonstrate the psoas muscle, and **c**, **f**, **i** demonstrate the aorta

For all anatomical structures, the noise magnitude was the lowest with DLR, followed by HIR, MBIR, and FBP. The detailed results were shown in Table 3 and Fig. 3. The NPS average spatial frequency (f_av_) values were higher using DLR than HIR, while FBP demonstrated the highest f_av_ values. The normalized NPS showed that for the aorta and psoas muscle, the noise on DLR and MBIR images consisted of more high-frequency components than HIR.

**Table 3:** Noise magnitude and average noise power spectrum (NPS) spatial frequency obtained for images reconstructed with FBP, HIR, MBIR, and DLR

Parameter	Anatomical structure	DLR	MBIR	HIR	FBP
Noise magnitude (HU)	Aorta	9.70 (1.14)	27.78 (4.61)	17.06 (2.64)	53.36 (8.13)
	Liver	10.32 (0.74)	27.11 (3.32)	18.12 (1.54)	49.02 (9.39)
	Psoas muscle	10.25 (1.16)	23.86 (2.86)	16.80 (2.06)	42.71 (6.32)
Average NPS spatial frequency (mm^−1^)	Aorta	0.27 (0.03)	0.29 (0.03)	0.24 (0.02)	0.33 (0.02)
	Liver	0.19 (0.02)	0.22 (0.02)	0.18 (0.01)	0.29 (0.01)
	Psoas muscle	0.25 (0.04)	0.28 (0.04)	0.24 (0.03)	0.33 (0.03)

The results of the detailed blur effect are listed in Table 4. The blur effect of DLR images was significantly lower on the edges, textured areas, or homogeneous areas than that of HIR (all *p* < 0.016). In the liver and psoas muscle, DLR had a blur effect value similar to that of FBP (*p* = 1, *p* = 0.9696, respectively), which means that both had similar sharpness. For larger arteries (aorta and femoral artery), the trend of blur effect from low to high was FBP, MBIR, DLR, and HIR, while the blur effect value of FBP and MBIR images were significantly lower than those of DLR and HIR (all *p* < 0.001). For the popliteal artery, the blur effect of the DLR was almost the same as that of the FBP (*p* = 1). The trend of blur effect from low to high for the whole section image (aorta level, femoral artery level, and popliteal artery level) was FBP, MBIR, DLR, and HIR, all significantly different from each other with pairwise comparison (all *p* < 0.001). Except at the femoral artery level, the blur effect of the MBIR image was consistent with that of FBP (*p* = 0.17).

**Table 4:** Objective sharpness analysis

Parameter	DLR	MBIR	HIR	FBP	p*
*Structures*					
Aorta	0.47 ± 0.05	0.39 ± 0.04*	0.50 ± 0.04*°	0.37 ± 0.04*˜	< 0.001
Femoral artery	0.39 ± 0.06	0.35 ± 0.06*	0.42 ± 0.06*°	0.35 ± 0.06*˜	< 0.001
Popliteal artery	0.29 ± 0.10	0.28 ± 0.09*	0.32 ± 0.10*°	0.29 ± 0.09°˜	< 0.001
Liver	0.30 ± 0.05	0.26 ± 0.02*	0.33 ± 0.03*°	0.30 ± 0.03°˜	< 0.001
Psoas muscle	0.33 ± 0.03	0.28 ± 0.03*	0.35 ± 0.03*°	0.32 ± 0.03°˜	< 0.001
*Whole section image*					
Aorta level	0.35 ± 0.03	0.29 ± 0.02*	0.36 ± 0.02*°	0.26 ± 0.02*°˜	< 0.001
Femoral artery level	0.31 ± 0.01	0.26 ± 0.01*	0.33 ± 0.01*°	0.26 ± 0.01* ˜	< 0.001
Popliteal artery level	0.31 ± 0.01	0.28 ± 0.01*	0.35 ± 0.01*°	0.33 ± 0.01*°˜	< 0.001

*DLR* deep learning–based reconstruction, *MBIR* model-based iterative reconstruction, *HIR* hybrid-iterative reconstruction, *FBP* filtered back projection

Pairwise comparisons showed significant differences from DLR (*), MBIR (°), and HIR (˜) (*p* < 0.05)

### Qualitative analysis

The higher the score is given by the radiologists, the better the image quality. The results of the qualitative evaluation are summarized in Table 5. Interreader reliability was good (coefficient: 0.872, *p* < 0.001). The image noise, subjective sharpness, and natural appearance at different levels of lower extremity arteries as well as soft tissues were evaluated. The noise of the lower extremity arteries from low to moderate with the four reconstruction algorithms is DLR, HIR, MBIR, and FBP. There were significant differences between the pairwise comparisons except for the comparison between HIR and MBIR (*p* = 1.00). Regarding subjective sharpness, the trend from better to moderate image quality is DLR, MBIR, HIR, and FBP. There were no significant differences between the pairwise comparison of DLR and MBIR (*p* = 0.049) or MBIR and HIR (*p* = 0.13). For a natural appearance, DLR received the highest score, and FBP received the lowest score. There was no significant difference in the pairwise comparison between DLR, MBIR, HIR, and FBP except for DLR and FBP at the popliteal artery (*p* = 0.011). For soft tissues, including liver and psoas muscle, the trend from high to low image quality was DLR, HIR, MBIR, and FBP for noise with a significant difference; DLR, MBIR, HIR and FBP for subjective sharpness, there was no significant difference between MBIR and HIR (*p* = 1.00); and DLR, HIR. For a natural appearance, there was no significant difference between MBIR and FBP (*p* = 0.32, *p* = 0.89, respectively), HIR and FBP (*p* = 0.19, *p* = 0.36, respectively) for liver and psoas muscle, nor was DLR and HIR for psoas muscle (*p* = 0.058).

**Table 5:** Qualitative analysis

Parameter	DLR	MBIR	HIR	FBP	*p*
*Aorta*					
Noise	4.98 [4.91–5.05]	3.94 [3.84–4.03]*	4.08 [4.00–4.16]*	3.19 [3.07–3.30]*°˜	< 0.001
Subjective sharpness	4.94 [4.87–5.01]	4.56 [4.41–4.71]*	4.23 [4.11–4.35]*	3.69 [3.55–3.82]*°˜	< 0.001
Natural appearance	4.23 [4.09–4.37]	3.95 [3.88–4.02]	4.00 [3.89–4.10]	3.85 [3.73–3.96]	< 0.001
*Femoral artery*					
Noise	4.90 [4.81–4.99]	3.92 [3.80–4.03]*	4.06 [3.97–4.16]*	3.00 [2.94–3.06]*°˜	< 0.001
Subjective sharpness	4.94 [4.87–5.00]	4.52 [4.37–4.67]*	4.14 [4.04–4.25]*	3.50 [3.35–3.65]*°˜	< 0.001
Natural appearance	4.28 [4.13–4.43]	4.00 [3.93–4.07]	3.97 [3.88–4.07]	3.77 [3.63–3.91]*	< 0.001
*Popliteal artery*					
Noise	4.85 [4.75–4.96]	3.90 [3.76–4.03]*	4.06 [3.97–4.16]*	3.10 [3.00–3.21]*°˜	< 0.001
Subjective sharpness	4.79 [4.67–4.91]	4.56 [4.42–4.71]	4.08 [3.98–4.18]*°	3.73 [3.60–3.86]*°	< 0.001
Natural appearance	4.08 [3.99–4.16]	3.97 [3.92–4.03]	3.95 [3.84–4.05]	3.87 [3.76–3.98]	0.031
*Liver*					
Noise	4.81 [4.70–4.93]	3.02 [2.97–3.06]*	3.98 [3.94–4.02]*°	3.02 [2.98–3.06]*	< 0.001
Subjective sharpness	4.60 [4.46–4.74]	3.98 [3.85–4.11]*	4.08 [4.00–4.16]*	3.15 [3.04–3.25]*°˜	< 0.001
Natural appearance	4.67 [4.51–4.82]	3.28 [3.13–3.43]*	4.00 [3.93–4.07]*°	3.62 [3.45–3.78]*	< 0.001
*Psoas muscle*					
Noise	4.90 [4.81–4.99]	3.1 [3.00–3.21]*	4.00 [3.92–4.08]*°	2.94 [2.87–3.01]*	< 0.001
Subjective sharpness	4.92 [4.84–5.00]	4.15 [4.03–4.27]*	4.08 [4.00–4.16]*	3.1 [3.01–3.19]*°˜	< 0.001
Natural appearance	4.46 [4.30–4.62]	3.46 [3.30–3.63]*	3.97 [3.88–4.07]°	3.67 [3.51–3.82]*	< 0.001

Estimated marginal means for every quality parameter, reconstruction algorithm and pairwise comparison significance are given, with 95% confidence intervals in brackets

*DLR* deep learning–based reconstruction, *MBIR* model-based iterative reconstruction, *HIR* hybrid-iterative reconstruction, *FBP* filtered back projection

Pairwise comparisons showed significant differences from DLR (*), MBIR (°), and HIR (˜) (*p* < 0.05)

### Diagnostic accuracy

**Fig. 4:**
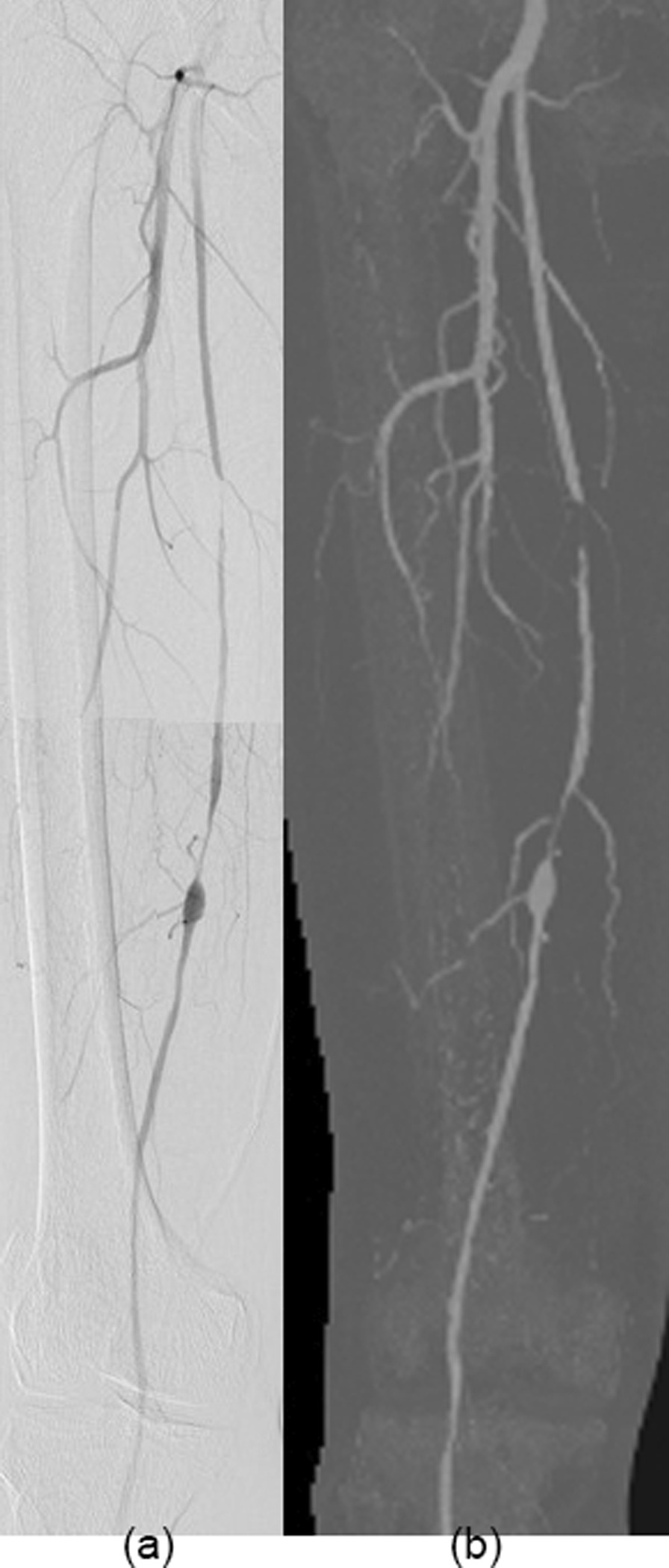
A 67-year-old male with intermittent claudication of the right lower limb for 1 month. There was occlusion and significant stenosis in the proximal and middle superficial femoral arteries, respectively. An aneurysmal dilatation was found immediately after significant stenosis. DSA was performed within twenty-nine days. Panel **a** shows the DSA image and Panel **b** shows the maximum intensity projection CTA image

**Fig. 5:**
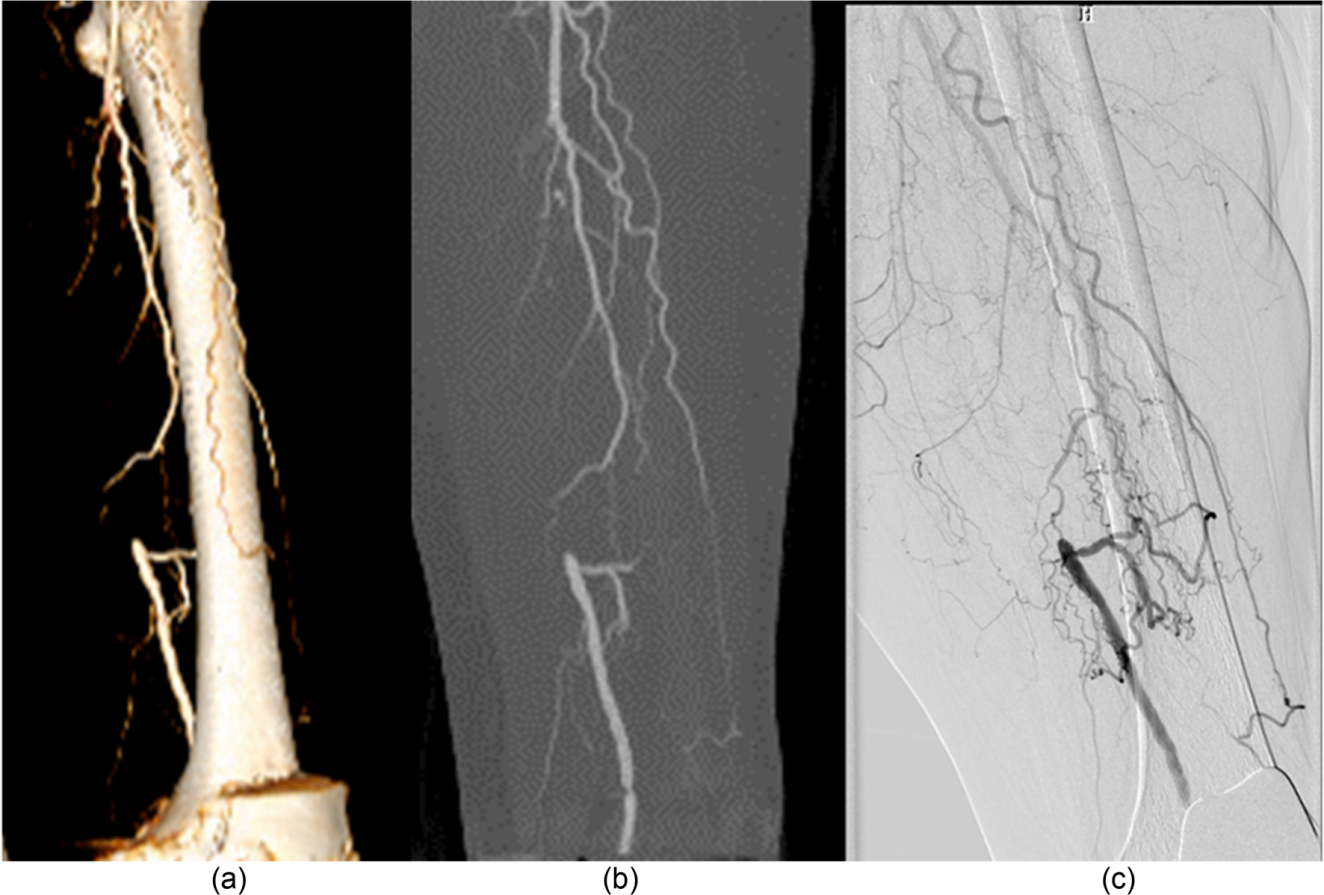
A 70-year-old female with left gangrenous toe for 3 weeks. There was an occlusion in the left proximal superficial femoral artery. The collateral artery in the distal patent superficial femoral artery was found. Panel **a** shows the volume rendering CTA image, panel **b** shows the maximum intensity projection CTA image, and panel **c** shows the DSA image

**Fig. 6:**
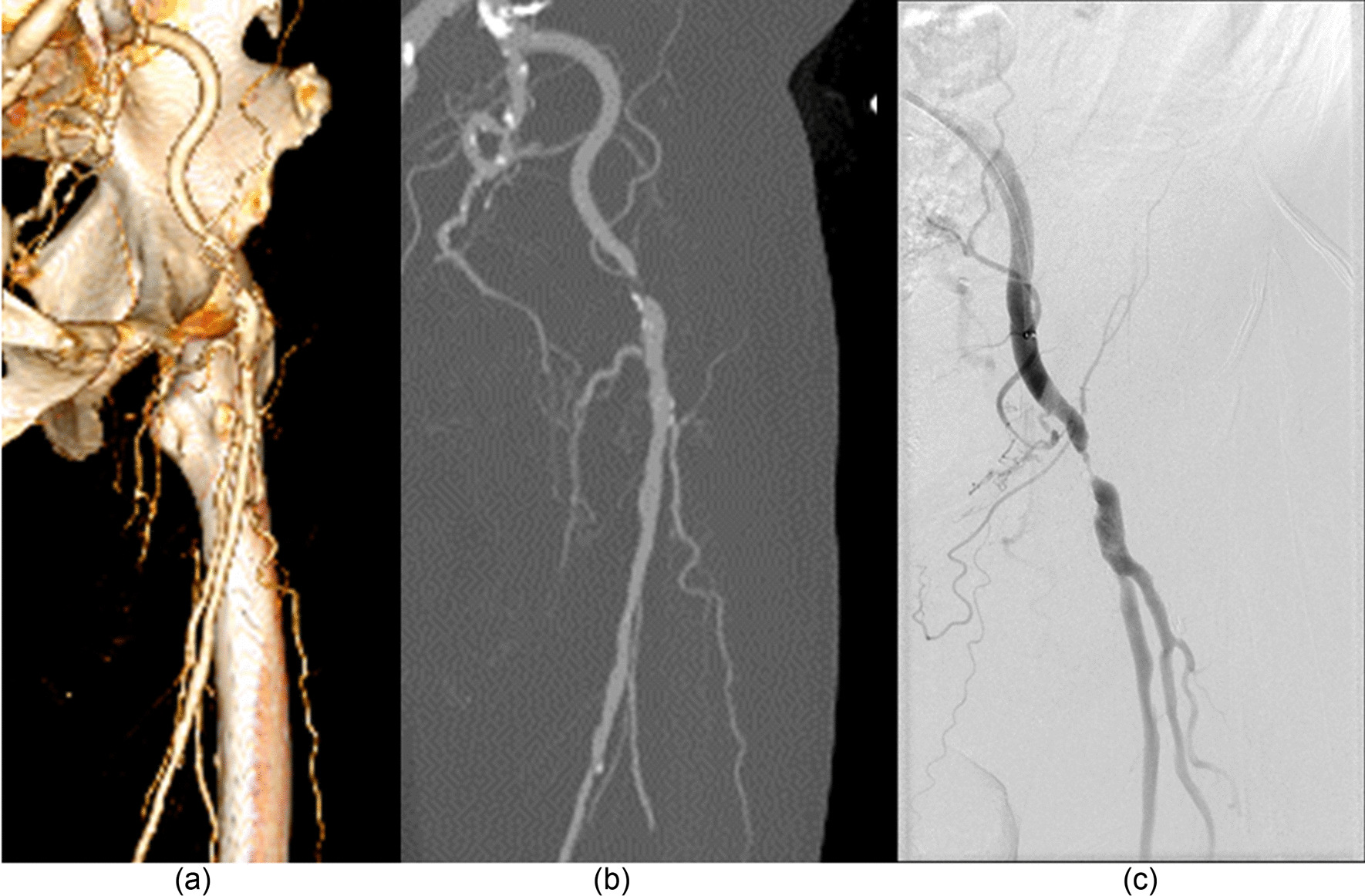
A 77-year-old male with intermittent claudication of both lower limbs for 3 months. There was significant stenosis in the left external iliac artery. Panel **a** shows the volume rendering CTA image, panel **b** shows the maximum intensity projection CTA image, and panel **c** shows the DSA image

Using DSA as a reference (Figs. 4, 5, 6), the sensitivity, specificity, positive predictive value (PPV), and negative predictive value (NPV) for the detection of stenosis by run-off CTA using the DLR, MBIR, HIR, and FBP reconstruction algorithm are shown in Table 6. The sensitivity from high to low was DLR, MBIR, HIR, and FBP (98.4%, 96.7%, 95.1%, and 93.4%, all *p* > 0.05), and the specificity from high to low was DLR, HIR, MBIR, and FBP (97.2%, 96.5%, 95.7%, and 93.7%, all *p* > 0.05).

**Table 6:** Diagnostic accuracy of the four reconstruction algorithms

	DLR	MBIR	HIR	FBP
Sensitivity	98.4% (95% CI, 90.0–99.9%)	96.7% (95% CI, 87.6–99.4%)	95.1% (95% CI, 85.4–98.7%)	93.4% (95% CI, 83.3–97.9%)
Specificity	97.2% (95% CI, 92.5–99.1%)	95.7% (95% CI, 90.6–98.3%)	96.5% (91.6–98.7%)	93.7% (95% CI, 88.0–96.9%)
PPV	93.8% (95% CI, 84.0–98.0%)	90.8% (95% CI, 80.3–96.2%)	92.1% (95% CI, 81.7–97.0%)	86.4% (95% CI, 75.2–93.2%
NPV	99.3% (95% CI, 95.5–100.0%)	98.6% (95% CI, 94.3–99.7%)	97.9% (95% CI, 93.4–99.4%)	97.1% (95% CI, 92.2–99.1%)

*PPV* positive predictive value, *NPV* negative predictive value, all *p* > 0.05

## Discussion

This study evaluated the image quality and diagnostic accuracy in lower extremity CTA reconstructed by DLR. For conventional quantitative image quality, the noise magnitude was lowest in DLR images and resulted in the highest SNR and CNR. There were more high-frequency components in the noise of DLR and MBIR images than that of HIR. As a complement to conventional objective image quality evaluation, the blur metric which evaluates the absolute quantitative value of the blur effect, showed that DLR preserves image sharpness compared to HIR. For subjective assessment, DLR received the highest score based on image noise, subjective sharpness, and natural appearance. The diagnostic performance of DLR was the best among the four reconstruction algorithms.

This is the first study on the application of the DLR algorithm “AiCE” in lower extremity CTA. There are several studies on other anatomic parts and the phantom. The results of this study are consistent with those of previous studies on cerebral non-contrast CT [12], coronary CTA [20], pulmonary CTA [21] abdominal CT [11, 22], and phantom [8]. The image noise, CNR, and/or SNR of DLR were improved compared with those of HIR and/or MBIR, while none of the previous studies compared DLR with FBP in vivo or discussed the blur metrics of sharpness.

Another DLR named “TrueFidelity” was explored by Park et al. on the application of lower extremity CTA with the discussion of blur metrics [14]. The CNR and SNR were higher in the DLR than in the low blending factor HIR, and the blur metrics increased as the strength of the DLR was improved. However, they did not evaluate MBIR and FBP at the same time.

In our study, DLR was compared with all three kinds of other reconstruction methods, MBIR, HIR, and FBP. In general, the objective image quality, including SD, CNR, and SNR, was best in DLR for both vascular lumen and soft tissues. Based on the subjective performance of the four reconstruction algorithms, radiologists ranked the highest score for DLR for both image noise, subjective sharpness, and natural appearance. This is consistent with other previous studies in which DLR achieved a better subjective score than the MBIR and/or HIR [11, 12, 20, 22]. The diagnostic performance of lower extremity runoff CTA with routine image reconstruction was excellent [1, 23], as was DLR in this study.

No-reference blur assessment in this study showed that DLR was better than Hybrid-IR with less blur effect and closer to MBIR. The possible reason might be that the training set of DLR was high-quality MBIR, which might preserve a similar sharpness property. MBIR had higher sharpness in this study, possibly due to the sharp kernel that we applied, which improved the image sharpness. FBP showed the best sharpness, which might be due to the highest image noise. Since the blur effect metric in this study was not focused on dedicated anatomical parts or edges, the location of the rectangular parts of the image was approximately selected to evaluate the general blur effect with a multiple-filtering process and comparison. This is not consistent with the subjective perception of image sharpness and is directly related to diagnostic performance. Even so, DLR showed better sharpness than HIR.

The application of lower extremity runoff CTA is increasingly popular; however, at the same time, the radiation dose related to the scan protocol and large scan range for the lower extremities is underexplored. In previous studies, the image quality of DLR was even better despite a dose reduction of 20%-30% compared to HIR images with a reference dose [20–22] in cardiac CTA, abdominal CT, and pulmonary CTA, and the reduction can even reach 50% in pediatric CT [24]. Although the radiation dose was not reduced in this study, the excellent image quality performance of DLR provides the potential application of DLR in low-dose lower extremity CTA.

Compared to HIR, the CNR of MBIR was lower in all locations, and SNR was lower in the aorta and soft tissues. The results were different from the phantom study [25] and cardiac CT [26]. The subjective score of MBIR was worse for noise and better for the structure's natural appearance compared with HIR. For MBIR, the objective score of noise was similar to the subjective noise result of this study, in which noise of MBIR was higher in the aorta, liver, and psoas muscle, and was consistent with Akagi et al. [22] and Bornet et al.’s study [11]. In previous studies, the natural appearance of cerebral [12] and abdominal [11] CT was slightly worse in MBIR, and the result was different from that in this study, which may be caused by different study anatomic areas and reconstruction parameters, such as kernel selection. To keep the parameters of the DLR in line with the MBIR, we used the body sharp kernel in this study. Although the so-called “sharp” kernels usually maintain high spatial resolution, they also elevate the image noise [27, 28].

There are several limitations to this study. First, we only compared DLR with MBIR, HIR, and FBP in routine radiation doses. According to previous studies, the image quality of DLR at a low radiation dose was comparable to that of HIR [21, 24, 29]. While there was no study on a low radiation dose of DLR in lower extremity run-off CTA, further study needs to be done. Second, “body sharp” was selected as the kernel of DLR and MBIR in this study, which might increase the noise magnitude of MBIR and lower its CNR in all locations, and SNR in the aorta and soft tissues. The influence of kernels in the comparison of DLR with other reconstruction algorithms should be discussed in further studies. Third, findings in this study may be valid no longer when the CTA is performed by newly proposed sparse-view CT imaging combined with a novel deep learning method [30–32], and further investigations are needed. Fourth, our study is limited by the small number of patients who underwent DSA, DLR diagnostic performance analysis sub-grouped by the location of the lesion was not performed.

## Conclusions

In conclusion, compared to other routine reconstruction methods, DLR showed better objective image quality in terms of CNR, SNR, and image noise for both vessels and soft tissues, as did the subjective image quality score in terms of noise, subjective sharpness, and natural appearance. The diagnostic accuracy of lower extremity CTA with DLR was the best among four reconstruction algorithms.

## Data Availability

The datasets used and/or analyzed during the current study are available from the corresponding author upon reasonable request.

## Abbreviations

PAD: Peripheral artery disease
CVD: Cardiovascular disease
CTA: Computed tomography angiography
FBP: Filtered back projection
HIR: Hybrid iterative reconstruction
MBIR: Model-based iterative reconstruction
DLR: Deep learning-based CT reconstruction
AiCE: Advanced intelligent clear-IQ engine
DCNN: Deep convolutional neural network
CTDIvol: CT dose index volume
DLP: Dose-length product
DSA: Digital subtraction angiography
ROI: Region of interest
SD: Standard deviation
CNR: Contrast-to-noise ratio
SNR: Signal-to-noise ratio
NPS: Noise power spectrum NPS
DICOM: Digital imaging and communications in medicine
BMI: Body mass index
